# Interpretation of Data Workshop in the Pediatric Preclerkship Educational Exercises (PRECEDE) Curriculum

**DOI:** 10.15766/mep_2374-8265.10496

**Published:** 2016-11-10

**Authors:** Eric Balighian, Michael Barone, David Cooke, Stacy Cooper, Robert Dudas, Emily Frosch, W. Christopher Golden, Justin Jeffers, Rosalyn Stewart

**Affiliations:** 1 Assistant Professor of Pediatrics, Johns Hopkins University School of Medicine; 2 Associate Professor of Pediatrics, Johns Hopkins University School of Medicine; 3 Associate Professor of Psychiatry, Division of Child and Adolescent Psychiatry, Johns Hopkins University School of Medicine; 4 Associate Professor of Medicine and Pediatrics, Johns Hopkins University School of Medicine

**Keywords:** Interpretation, Clinical Clerkship, Pediatrics, PRECEDE, Data

## Abstract

**Introduction:**

The PRECEDE (preclerkship educational exercises) curriculum was developed by the Johns Hopkins Pediatrics Clerkship with the primary goal of providing students with experiential, explicit, and standardized instruction in essential pediatric clinical skills to better prepare them for their clerkships. This data interpretation workshop is one of a series of modules within the PRECEDE curriculum presented just prior to the clerkship.

**Methods:**

This 2-hour workshop divides medical students into small groups of four to six students, each group with a faculty facilitator. Three clinical vignettes are presented to the student groups. For each vignette, students participate in a guided discussion to develop an interpretation and understanding of what is often misinterpreted pediatric information, such as developmental milestones, vital signs, and laboratory values.

**Results:**

From the perspectives of students and instructors, the implementation of this new module was very successful in terms of its delivery of educational content and its organizational structure. Of 229 students who participated in the session and completed surveys, 77% strongly agreed the session was educationally valuable.

**Discussion:**

By establishing important basic skills of correct pediatric data interpretation, students may be better equipped to develop appropriate differential diagnoses, assessments, and plans of care for patients.

## Educational Objectives

By the end of this session, pediatric clerkship students will be able to:

1. Review the variability in normal child development, vital signs, laboratory values, and radiographic findings.
2. Use pediatric reference materials for accessing norms of pediatric data.
3. Develop differential diagnoses for common pediatric presentations based on age-specific data.

## Introduction

The Johns Hopkins Pediatrics Clerkship developed the PRECEDE (preclerkship educational exercises) curriculum with the primary goal of providing students with experiential, explicit, and standardized instruction in essential pediatric clinical skills to better prepare them to incorporate these skills during their clerkship. The PRECEDE curriculum may be implemented in part or in its entirety, depending on the needs of the training program, and can be modified according to the availability of instructors and pediatric patient populations. This data interpretation workshop is one of a series of modules offered during the clerkship.

The Council on Medical Student Education in Pediatrics’ curricular objectives^1^ state that all students should be able to:

1. Interpret the results of commonly used diagnostic tests.
2. Identify variations in vital signs based on age of the patient, presence or absence of disease, and testing modalities.
3. Accurately identify and interpret major developmental milestones of the neonate, infant, toddler, school-age child, and adolescent.
4. Demonstrate an ability to generate an age-appropriate differential diagnosis and problem list.
5. Inform the health care team of the relevant thought process and decisions.

This workshop emphasizes these objectives. The development of this session was prompted by a needs assessment that asked students early in their clerkships, “What clinical skills or knowledge specific to this clerkship do you wish you had been explicitly taught, with opportunity for practice and feedback, before you started to see patients on the pediatric clerkship?”^2^ This local needs assessment was further refined by discussions with clerkship directors from other disciplines at our institution to avoid redundancies in the delivery of clinical skills instruction.^2^

This module is unique in that it meets an identified student need to learn pediatric normative data in context. This session employs case-based learning and small-group facilitation, both well-known interactive strategies. Rather than focusing comprehensively on a single topic area, the module provides students with guiding age-based principles and familiarity with commonly used resources. This allows students to build their interpretation skills throughout their clinical experience.

Interpretation of clinical data is a necessary skill in clinical reasoning. Pediatric data are particularly subject to misinterpretation due to the variability of what is normal depending on age. For example, a hemoglobin level of 9.9 grams per deciliter may be falsely considered low in a 2-month-old when it is in fact within normal limits. Discussing common examples like this helps students understand the important concept of correctly interpreting the variability of data in children. If data are incorrectly interpreted as abnormal, then incorrect assessments, differential diagnoses, and potentially harmful testing may commence. If data are incorrectly interpreted as normal, when truly abnormal, then problems and diseases may be missed. Students also begin to discover where to reference appropriate resources when interpreting data. The student groups engage in active participation, which stimulates critical thinking and may be linked to gains in knowledge.

This data interpretation workshop is intended to be conducted on the first day of the PRECEDE curriculum. The PRECEDE curriculum is intended to span the 3 days prior to the actual pediatric clinical clerkship and then 2 days of additional workshops in the middle of the clerkship. This workshop is best situated before students actually start clinical rotations. If it is logistically impossible to conduct the workshop prior to the start of the rotation, conducting it in the middle of the clerkship would still be beneficial. The skills developed during the workshop are essential for the clinical care of children, and students with some clinical experience may enhance discussion and interaction in the workshop.

## Methods

The best location for this workshop is a large room containing at least one table per small group. The duration of the workshop should be about 2 hours. The room should have the ability to view PowerPoint presentations.

The workshop is best done with one clinical facilitator for each small group. One lead facilitator navigates through the PowerPoint presentation (Appendix B); he or she can also facilitate a group as well. The other facilitators lead each student group of four to six students. The lead facilitator navigates the PowerPoint and stops when there are pediatric data for the small groups to interpret and discuss. Each group should have a facilitated discussion about the data for several minutes. There should be texts or handbooks for students, such as the *Harriet Lane Handbook*^3^ and the *Bright Futures* pocket guide,^4^ available to each small group. Other resources that may help are textbooks with information about common pediatric diseases,^5^ textbooks with pictures of rashes and conditions,^6^ and textbooks that can help generate differential diagnoses.^7^ Students should be encouraged to use the resources available, as well as others they may already have access to, such as the internet. If students use other resources, they should be encouraged to consider the integrity and factual content of those resources.

After the small-group discussion, the lead facilitator summarizes important points to all groups. The lead facilitator also asks groups and individuals what interpretations and discussion they had during the small-group session. The lead facilitator then continues with the slide show until the next data are presented for group discussion. This flow continues until the slide-show presentation is complete. See the Figure for a visualization of the course flow.

**Figure.**
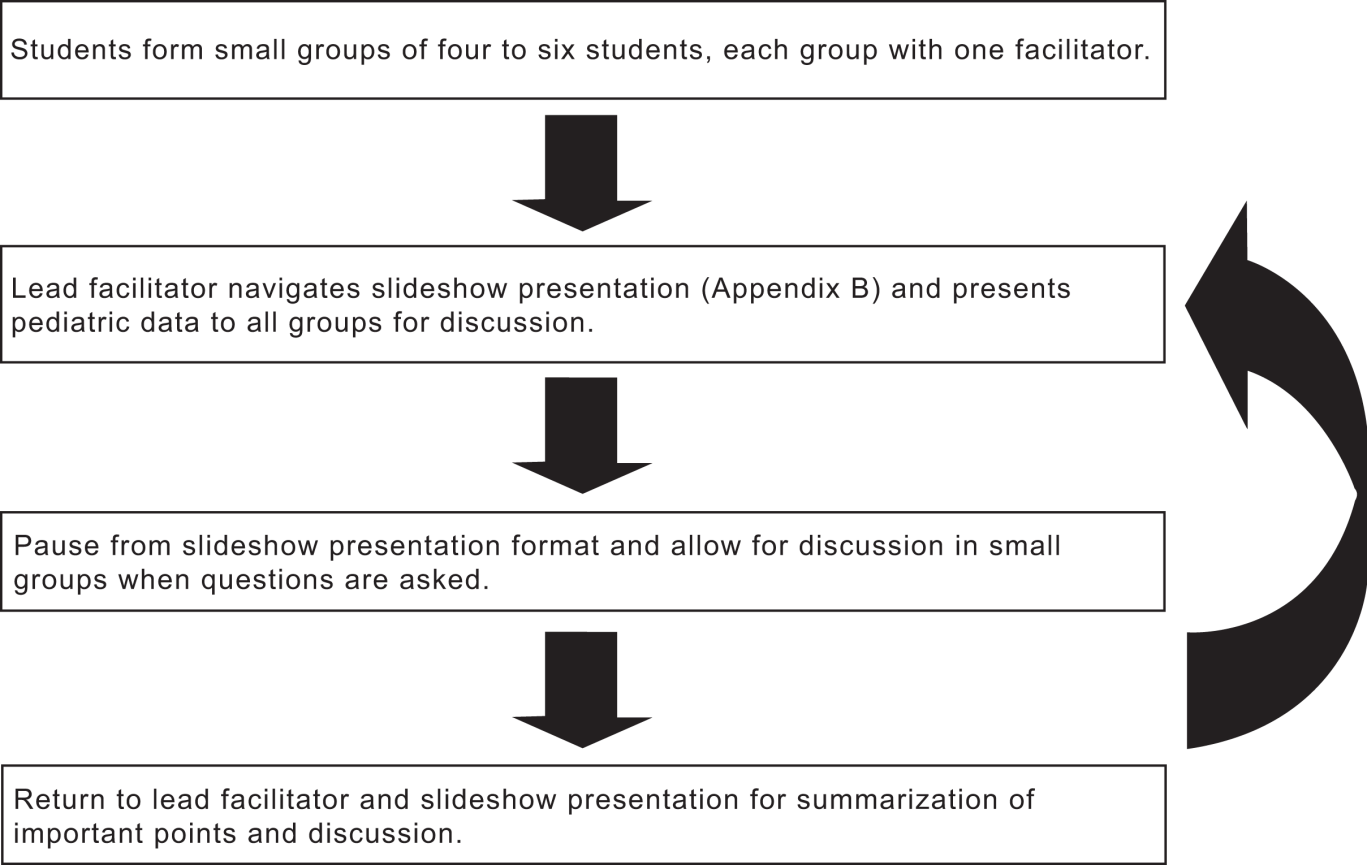
Course flow.

The lead facilitator should refer to the PowerPoint Guide for Lead Facilitator (Appendix A) while using the slide-show presentation.

The small-group facilitators should refer to the Small Group Facilitator Guide (Appendix C) when facilitating discussion during small-group sessions.

## Results

From the perspectives of students and instructors, the implementation of this new module was very successful in terms of its delivery of educational content and its organizational structure. In order to measure the impact of the new curriculum on medical students in the course, students completed surveys that were analyzed for the purposes of curriculum improvement. Between 2010 and 2012, 229 students completed surveys. Each survey item had four response options the students could choose: strongly disagree, disagree, agree, and strongly agree. All students agreed that the session was educationally valuable (see the Table). The great majority of students strongly agreed that the session increased their skills and would improve their ability to function on the wards. The survey item that had the largest proportion of students strongly agreeing was that active student participation was encouraged.

**Table.** Results of the Student Surveys (*N* = 229)

Survey Item	Percent Agree	Percent Strongly Agree
This session met the stated objectives.	28	72
This session increased my skills related to this topic.	27	73
Having had this session will improve my ability to function as a clinical clerk on the wards.	24	76
This session was educationally valuable.	23	77
The instructors provided a supportive learning environment.	17	83
Active student participation was encouraged.	14	86
The instructors enabled me to accomplish the learning objectives for the session.	17	83
The instructors’ teaching methods stimulated my own critical thinking.	18	82
	**Percent Rated as Good**	**Percent Rated as Excellent**
Please rate this session overall.	22	78

## Discussion

This session, focusing on building data interpretation skills in medical students at the start of their pediatric clerkship, has been very well received. The workshop's main objective of allowing students to recognize the variability of what is considered normal in children is accomplished through problem solving, pictures, and discussion. The old adage that “children are not little adults” is deeply explored, and students realize the tremendous variability among children.

The large-group format ensures important points and concepts are discussed with all students. The small-group discussions allow for students to problem solve by using past experiences and knowledge, both important concepts in adult learning theory.^8^ Another benefit of utilizing the small groups is that there is a wide variety of student experiences and knowledge with regard to interpreting pediatric clinical data. Having facilitators facilitate the discussion, but not rigidly direct it, is important.

On the clinical wards after participating in this workshop, students may be able to reference resources more frequently and may not make assumptions about data, though we do not have quantitative information to confirm this theory. Students appreciate knowing when to use references, which references to use, and how to use them.

Using realistic patient vignettes keeps the content interesting and provides examples that students may see during their pediatric experience. Pictures are engaging and provide an opportunity to discuss physical examination and clinical reasoning. For future versions, videos of patients would be even more engaging and interesting.

The development of the content, cases, pictures, and discussion points has evolved and been modified over the years based on student and facilitator feedback. After conducting approximately 25 of these sessions with over 500 students, we have learned that no session is the same. Discussion points and concepts are emphasized in varying degrees depending on the students’ interests, past experiences, and past knowledge.

## Appendices

A. PowerPoint Guide for Lead Facilitator.doc
B. Interpretation of Data.ppt
C. Small-Group Facilitator Guide.doc All appendices are peer reviewed as integral parts of the Original Publication.
