# Supplementary material for: Interpretation of Data Workshop in the Pediatric Preclerkship Educational Exercises (PRECEDE) Curriculum
Source: MedEdPORTAL. 2016 Nov 10;12:10496. doi: 10.15766/mep_2374-8265.10496 (PMC6440424; doi:10.15766/mep_2374-8265.10496)
Supplement: Supplementary file 1 — A. PowerPoint Guide for Lead Facilitator.doc B. Interpretation of Data.ppt C. Small-Group Facilitator Guide.doc [file mep-12-10496-s001.zip › A. PowerPoint Guide for Lead Facilitator.doc]

**PowerPoint Guide for Lead Facilitator**

**Interpretation of Data**

The lead facilitator should use the PowerPoint “Interpretation of Data” while conducting the workshop. The lead facilitator will navigate through the PowerPoint while pausing at certain slides with data, allowing small group discussion. After small group discussion, the lead facilitator should summarize important points outlined below, allowing large group discussion when appropriate.

This lead facilitator guide has the important principles that should be discussed. The small group guide has many more discussion topics and points. The important points discussed in this guide may or may not be discussed in each of the small groups as that would depend on the student’s spontaneous ideas, previous experiences, and interests.

The suggested questions, points, and discussion are outlined below for each slide.

**Slide 1:** Introductory Title Slide.

A common mistake made in pediatrics is when data (history, exam, lab, radiographs) is normal but interpreted as abnormal. And vice versa.

Healthcare providers that care for children must determine what is normal and what is abnormal by understanding the concept of the variability in children and then rely upon personal experience or references.

We will explore this concept and examples with 3 cases in 3 settings. The settings will be the outpatient clinic, the inpatient ward, and the emergency department.

**Slide 2 and 3:** Read goals and objectives.

**Slide 4**: The first patient is a 13 month old who presents to us at the outpatient primary care clinic. Read slide to large group.

**Slide 5**: Ask the small groups these questions:

Is not walking at 13 months normal? Is saying these three words normal? What references or tools can you use to help?

Allow the small group discussion for approximately 5 minutes.

After 5 minutes, have all small groups refocus on the lead facilitator. Ask a small group to summarize the discussion they had while answering the questions.

Summarize the following important points detailed on the next 3 slides.

**Slide 6 and78:** Note that difference between developmental screening and surveillance in children. Surveillance is flexible, longitudinal, and continuous process that skilled healthcare providers do almost subconsciously by observation and talking with parents. Screening, like other screening tests, is a deliberate assessment procedure designed to identify children who need more intense testing or assessment. ^1^

**Slide 8:** An example of a developmental screening tool some healthcare providers use is shown. This is a parent questionnaire. There are more developed questionnaires such as the Ages and Stages Questionnaires^2^.

Note some resources the students can use while assessing a child’s development when they see children soon in their outpatient clerkship rotation (Bright Futures Pocket Guidelines^3^, Harriet Lane Handbook (HLHB)^4^).

Note that considerable variability exists in achieving developmental milestones.

**Slide 9:** The same patient, a 13 month old, has this 2 cm rash on his right forearm.

**Slide 10:** Allow the small groups 5 minutes to discuss the following. Describe the rash and discuss the differential diagnosis.

**Slide 11**: After 5 minutes of discussion, ask a small group to summarize the description they came up with for this rash. Note the importance of using correct descriptive dermatologic terms when describing rashes. Note the difference between macules, patches, papules, and plaques. The benefit of using these dermatologic terms are one can communicate clearly with consultants or reference dermatologic texts when developing a differential diagnosis.

**Slide 12:** Note an example of atopic dermatitis in the flexural areas in a child. If time permits, can ask the large group for a description of this rash.

**Slide 13:** The same patient, a 13 month old male has these routine laboratory values obtained.

**Slide 14:** Ask the small groups to discuss the following questions. Are these labs normal and how can history or physical exam help. Students should access an appropriate resource like the HLHB. Allow a 7 minute discussion.

**Slide 15:** Ask a small group to summarize their discussion. The group should have concluded that this child has a microcytic anemia. They should have developed a differential diagnosis of microcytic anemia in a 13 month old that includes iron deficiency, alpha thalassemia, and lead toxicity. Note that an important part of evaluating laboratory values is referencing the age specific normal for each laboratory value. This slide portrays the significant variability of hemoglobin concentration and mean corpuscular volume according to age. The variability of laboratory values depending on age is an important concept for students to grasp as otherwise, one might attribute a value as abnormal when it really is normal, and vice versa.

**Slide 16:** We are now working on the inpatient wards, taking care of this 3 year old girl described in the slide.

**Slide 17:** The small groups should discuss the following. Interpretation of this 3 year old’s vital signs and what is the differential diagnosis. Allow 5-7 minutes for small group discussion.

After allowing for approximately 7 minutes of discussion, ask a small group to report out what they discussed and the differential diagnosis. This is a good time for a large group discussion on the potential diagnoses.

Another important concept is interpreting vital signs and the variability of vital signs according to age. Note that it would be extremely helpful for students to have an easy referenceable table with normal vital signs adjusted for age. This table can be found on the inner cover on most editions of the Harriet Lane Handbook, and students should find it to use when clinically caring for patients.

Another important concept to discuss, if time permits, is the differential diagnosis for tachycardia in children. One potential cause is anxiety. Having a large group discussion about overcoming anxiety in children when conducting an exam is helpful. Ask the large group to share ideas about how to distract children and allow children to be comfortable with healthcare providers. The facilitators in the room can also share experiences. This is an important discussion for students new to the pediatric clerkship.

**Slide 18:** Some healthcare providers may have opted to get a chest x-ray in this hospitalized child. Ask the large group, how are pediatric chest films different than adult chest film? Discussion points include children do not take a deep inspiratory film often, children are rotated often, the structures on film look different, and the thymus in children may be interpreted as abnormal when it is often a normal finding. The circle in the image on the right represents the thymic shadow which is normal.

**Slide 19:** We are now working in the outpatient emergency department, taking care of a 15 month old boy who has a seizure like episode at home. Have large group read slide.

**Slide 20:** Ask small groups to discuss the following questions. What else would they ask on history and what is differential diagnosis. Allow approximately 6-8 minutes for discussion in small groups.

After 6-8 minutes, ask a small group to report on the differential diagnoses they developed. Ask students in the large group to discuss the different diagnoses and they likelihood of each diagnosis.

**Slide 21:** This is a picture of the wrist of this child with a seizure like episode. Note the widening of the wrist. Ask the students what they think about this picture. Note the differential include a bone abnormality, for example a fracture, tumor, or rickets. Ask students if they would obtain further testing.

**Slide 22:** Radiography was obtained on this child’s wrists and hands on the right. Note the poor ossification of the bones and the cupping at the ends of the radius and ulna, suggestive of vitamin D deficiency or rickets. The chest film on the right shows a characteristic finding on the ribs called rosary beads also suggestive of rickets. Ask the students if they would obtain further testing.

**Slide 23:** Laboratory on this patient was obtained as depicted.

**Slide 24:** Ask students if these lab tests are normal. Allow approximately 4 minutes in the small groups for discussion.

After 4 minutes, ask a small group to report out their findings. Students should note the hypocalcemia, hypophosphatemia, and an elevated alkaline phosphatase. This further supports a diagnosis of Vitamin D deficiency which in children may cause rickets, if severe. Ask the students in the large group why this child may have developed rickets. The hypocalcemia, a consequence of rickets, caused the seizure in this child. Students should note that breastfed children should receive Vitamin D supplementation to prevent rickets^5^.

**Slide 25:** References slide

**References:**

1. Developmental Surveillance and Screening of Infants and Young Children. Committee on Children With Disabilities. [Pediatrics](http://pediatrics.aappublications.org/) Jul 2001, 108 (1) 192-195; DOI 10.1542/peds.108.1.192
2. Ages & Stages Questionnaires®, Third Edition (ASQ-3™), Squires & Bricker 2009 Paul H. Brookes Publishing Co.
3. Hagan JF, Shaw JS, Duncan P, eds. 2008. Bright Futures: Guidelines for Health Supervision of Infants, Children, and Adolescents, Third Edition. Pocket Guide. Elk Grove Village, IL: American Academy of Pediatrics.
4. Tschudy, Megan M.Arcara, Kristin M., eds. The Harriet Lane Handbook: A Manual For Pediatric House Officers. Philadelphia, PA : Mosby Elsevier, 2012. Print.
5. Adherence to Vitamin D Recommendations Among US Infants. Cria G. Perrine, Andrea J. Sharma, Maria Elena D. Jefferds, Mary K. Serdula, Kelley S. Scanlon. Pediatrics Apr 2010, 125 (4) 627-632; DOI: 10.1542/peds. 2009-2571
6. Behrman, Richard E, Robert Kliegman, and Waldo E. Nelson. *Nelson Essentials of Pediatrics*. Philadelphia: W.B. Saunders Co, 2002. Print.
7. Zitelli, B. J., In McIntire, S. C., & In Nowalk, A. J. (2012). *Zitelli and Davis' atlas of pediatric physical diagnosis*. Philadelphia, PA: Saunders/Elsevier.
8. Tunnessen, W. *Signs and Symptoms in Pediatrics*. Lipponcott Williams & Wilkins, 1999.
