# Supplementary material for: Interpretation of Data Workshop in the Pediatric Preclerkship Educational Exercises (PRECEDE) Curriculum
Source: MedEdPORTAL. 2016 Nov 10;12:10496. doi: 10.15766/mep_2374-8265.10496 (PMC6440424; doi:10.15766/mep_2374-8265.10496)
Supplement: Supplementary file 1 — A. PowerPoint Guide for Lead Facilitator.doc B. Interpretation of Data.ppt C. Small-Group Facilitator Guide.doc [file mep-12-10496-s001.zip › C. Small-Group Facilitator Guide.doc]

**Small Group Facilitator’s Guide**

**Interpretation of Data**

This document is intended for the small group facilitators. Ideally, each small group facilitator should read this document prior to the workshop.

Students should interpret data in the small group by thinking out loud, using past experiences and knowledge, and referencing appropriate resources. Facilitator will facilitate the discussion using the recommended questions, flow, and points below. This small group guide has many more principles, points, and discussion topics than the lead facilitator guide. Not all these points need to be discussed. The discussion should be tailored to each student group’s ideas and thoughts. The lead facilitator guide has important principles that should be discussed with the students during the large group time so do not worry about covering everything in the small groups.

**Tips for Facilitators leading these small groups**

When questions are asked of the students for each below discussion, facilitators should allow students an initial 2-3 minutes to use the resources provided or other resources they might use to find answers. Facilitators should also allow and encourage the students to discuss among themselves their thoughts and answers. If the students are having a difficult time initially, then the facilitator can take a more proactive role in showing them where to look up information and starting the discussion. Facilitators should allow students to even make mistakes at times and see if other students can correct. If no one corrects them, then the facilitator should, at some point, respectfully correct mistakes. In final minutes of the discussion, the facilitator should summarize the important points and concepts of each discussion especially if the students did not discuss them.

**Small Group Discussion 1: Developmental Milestones**

Is not walking at 13 months normal? Is saying three words normal? What references or tools can you use to help? What else can you ask on history to help?

(Questions on Slide 5)

(5 minute discussion)

Allow students to think out loud and debate what they know from past knowledge and experiences. Ask the students what other information they want to know while assessing this child. Appropriate answers include, “what else is the child doing, developmentally?”

Prompt students to refer at the Denver Developmental Milestone Diagram in the Harriet Lane Handbook (HLHB)^1^ or some other reference. Note the four areas of development that this particular reference uses (Language, Social, Gross motor, and Fine motor). There are several screening tools clinicians can use including Bright Futures Pocket Guide from the American Academy of Pediatrics (AAP)^2^. Having these references as examples would be helpful for students to explore.

If time permits, mention the AAP recommendation that children be formally screened for developmental delay at periodic intervals and when developmental concerns are raised by the parent or provider during routine developmental surveillance. The AAP recommends that infants and young children undergo formal developmental screening at the following ages: 9 months, 18 months, 24 or 30 months.

**Small Group Discussion 2: Physical Exam finding of a rash**

Describe the rash. What is the differential diagnosis?

(Questions on Slide 10)

(5 minute discussion)

Students should describe this rash trying to use correct dermatological terms including location, size, primary lesion, secondary lesions, color, and other features.

Based on this description or just by previous experience, have the students develop a differential diagnosis. Ask the students how they generated that differential diagnosis. Note that by good description of the rash, the student could reference a more experienced clinician or dermatology textbook by describing the rash well for identification. Discuss the importance of correctly describing skin findings using medical terms.

**Small Group Discussion 3: Laboratory Blood cell Indices**

Are these labs normal? How can history or physical exam help?

(Questions on slide 14)

(7 minute discussion)

Have students think out loud. Mention that the HLHB has normal values for the blood cell indices in the Hematology chapter. Students, while referencing the HLHB, should recognize the patient’s hemoglobin and MCV are more than 2 standard deviations below the mean. The students should recognize that the patient has a microcytic anemia. Ask the students to develop a differential diagnosis. The most common causes for this age include iron deficiency or alpha thalassemia trait. Mention that the AAP recommends screening for anemia and (depending on the patient’s home zip code) lead poisoning at one year and two years. Mention referencing Bright Futures for recommended screening at each age. Ask what additional information the students would want to know to differentiate the diagnoses they developed. Emphasize the importance of a diet history, past medical history, and the physical examination.

Mention that iron deficiency can occur from drinking too much cow’s milk as the iron is not as bioavailable as breastmilk or iron obtained from different foods.

**Small Group Discussion 4: Interpreting vital signs**

Interpret this child’s vital signs. What is the differential diagnosis?

(Questions on Slide 17)

(7 minutes)

Have students think out loud and discuss. They should reference age-specific pediatric normal vital signs, commonly found on the inside front cover of the HLHB. Can note that the only normal sign is the blood pressure. Note that for blood pressure, height, age, and gender influence the normal values. Ask them to develop a differential diagnosis for tachycardia in children. The differential should include dehydration, fever, pain, anxiety, and infection. Facilitate the discussion about the differential diagnosis which should include pneumonia, bronchiolitis, or an asthma exacerbation.

**Small Group Discussion 5: Pediatric symptoms of seizure-like episode**

What else would you want to know on history? What is the differential diagnosis?

(Questions on Slide 20)

(8 minutes)

Have students think out loud and generate discussion. Have them develop a differential diagnosis for seizure-like episode (make sure you say seizure-like episode and not seizure). The differential for seizure-like episode in this age includes toxic ingestion, febrile seizure, seizure disorder, head injury, abuse, brain lesion, and electrolyte abnormality. Ask, how would the students differentiate their differential diagnosis from each other?

Can note that the low weight and height percentiles are non-specific but should raise suspicion that a more chronic process is in effect but with relative head size sparing.

**Small Group Discussion 6: Interpreting laboratory values**

Are these labs normal?

(Slide 24)

(4 minutes)

Students should again reference the HLHB for age norms. Note the patient has hypocalcemia, hypophosphatemia, and an elevated alkaline phosphatase. What is the differential diagnosis now? How would children become Vitamin D deficient? The hypocalcemia, a consequence of rickets, may have caused the seizure in this child. Discuss how vitamin D is the only supplement recommended by the AAP for exclusively breastfed infants.

**References**

1. Tschudy, Megan M.Arcara, Kristin M., eds. The Harriet Lane Handbook: A Manual For Pediatric House Officers. Philadelphia, PA : Mosby Elsevier, 2012. Print.
2. Hagan JF, Shaw JS, Duncan P, eds. 2008. Bright Futures: Guidelines for Health Supervision of Infants, Children, and Adolescents, Third Edition. Pocket Guide. Elk Grove Village, IL: American Academy of Pediatrics.
3. Behrman, Richard E, Robert Kliegman, and Waldo E. Nelson. *Nelson Essentials of Pediatrics*. Philadelphia: W.B. Saunders Co, 2002. Print.
4. Zitelli, B. J., In McIntire, S. C., & In Nowalk, A. J. (2012). *Zitelli and Davis' atlas of pediatric physical diagnosis*. Philadelphia, PA: Saunders/Elsevier.
5. Tunnessen, W. *Signs and Symptoms in Pediatrics*. Lipponcott Williams & Wilkins, 1999.
